# Supplementary material for: Relationship between Exercise Test Parameters, Device-Delivered Electric Shock and Adverse Clinical Events in Patients with an Implantable Cardioverter Defibrillator for Primary Prevention
Source: J Pers Med. 2023 Mar 28;13(4):589. doi: 10.3390/jpm13040589 (PMC10143101; doi:10.3390/jpm13040589)
Supplement: Supplementary file 1 [file jpm-13-00589-s001.zip › jpm-2286333-Supplementary Table S1.pdf]

Supplementary Table S1: Exercise Test Parameters

Respiratory Exchange Ratio	1.2 (1.0-1.3)
Resting heart rate, bpm	70.0 (59.0-81.0)
Maximal heart rate, bpm	126.0 (105.0-147.0)
Resting systolic blood pressure, mmHg	110.0 (94.8-125.2)
Maximal systolic blood pressure, mmHg	140.0 (116.7-163.3)
Maximum power developed, Watts	88.0 (52.3-123.7)
Maximum power developed, METS	4.5 (3.2-5.8)
T1/2 VO2	103.0 (49.5-156.5)
1st ventilatory threshold, mL/min/kg	9.6 (6.9-12.3)
Circulatory power	2265.5 (936.2-3201.7)
Peak VO2, mL/min/kg	16.2 (11.6-20.8)
Peak VO2, % of theoretical maximum	66.5 (47.9-85.1)
VE/VCO2 Slope	35.0 (28.1-41.9)

Values are presented as median (quartile 1, quartile 3)

METs, Metabolic Equivalent of Task; T1/2 VO2, VO2 half-time; VE, ventilation minute; VCO2, carbon dioxide production; VO2, oxygen uptake.
